# Alert but less alarmed: a pooled analysis of terrorism threat perception in Australia

**DOI:** 10.1186/1471-2458-11-797

**Published:** 2011-10-12

**Authors:** Garry Stevens, Kingsley Agho, Melanie Taylor, Alison L Jones, Jennifer Jacobs, Margo Barr, Beverley Raphael

**Affiliations:** 1 School of Medicine, University of Western Sydney, Building EV, Parramatta Campus, Locked Bag 1797, Penrith NSW DC2751, Australia; 2 Graduate School of Medicine, Building 28, University of Wollongong, NSW, 2522, Australia; 3 Centre for Epidemiology and Research, New South Wales Department of Health, 73 Miller Street, North Sydney, NSW, 2060, Australia

**Keywords:** Terrorism, threat perception, habituation, ethnicity, education, psychological distress

## Abstract

**Background:**

Previous Australian research has highlighted disparities in community perceptions of the threat posed by terrorism. A study with a large sample size is needed to examine reported concerns and anticipated responses of community sub-groups and to determine their consistency with existing Australian and international findings.

**Methods:**

Representative samples of New South Wales (NSW) adults completed terrorism perception questions as part of computer assisted telephone interviews (CATI) in 2007 (N = 2081) and 2010 (N = 2038). Responses were weighted against the NSW population. Data sets from the two surveys were pooled and multivariate multilevel analyses conducted to identify health and socio-demographic factors associated with higher perceived risk of terrorism and evacuation response intentions, and to examine changes over time.

**Results:**

In comparison with 2007, Australians in 2010 were significantly more likely to believe that a terrorist attack would occur in Australia (Adjusted Odd Ratios (AOR) = 1.24, 95%CI:1.06-1.45) but felt less concerned that they would be directly affected by such an incident (AOR = 0.65, 95%CI:0.55-0.75). Higher perceived risk of terrorism and related changes in living were associated with middle age, female gender, lower education and higher reported psychological distress. Australians of migrant background reported significantly lower likelihood of terrorism (AOR = 0.52, 95%CI:0.39-0.70) but significantly higher concern that they would be personally affected by such an incident (AOR = 1.57, 95%CI:1.21-2.04) and having made changes in the way they live due to this threat (AOR = 2.47, 95%CI:1.88-3.25). Willingness to evacuate homes and public places in response to potential incidents increased significantly between 2007 and 2010 (AOR = 1.53, 95%CI:1.33-1.76).

**Conclusion:**

While an increased proportion of Australians believe that the national threat of terrorism remains high, concern about being personally affected has moderated and may reflect habituation to this threat. Key sub-groups remain disproportionately concerned, notably those with lower education and migrant groups. The dissonance observed in findings relating to Australians of migrant background appears to reflect wider socio-cultural concerns associated with this issue. Disparities in community concerns regarding terrorism-related threat require active policy consideration and specific initiatives to reduce the vulnerabilities of known risk groups, particularly in the aftermath of future incidents.

## Background

Terrorism affects populations primarily through the threat of its occurrence. While those directly exposed may suffer profound physical and psychological effects, the conveyed imagery of such incidents is intended to undermine the working assumptions of a populace: safety in public places, during routine travel and the predictability of risk [1]. Understanding population effects and those sub-groups with potentially greater vulnerability are critical elements of a comprehensive counter-terrorism strategy [1,2].

A principal reason terrorism is effective at the societal level is that it draws together two key elements known to inflate risk estimates; 'dread risk', which involves threats seen as catastrophic, uncontrollable and arbitrary in their impacts, and the 'unknown risk' of hazards that may not be observable or where the mechanism of injury is unfamiliar [3]. Threats with such features are typically overestimated in terms of their probability and risk of personal harm, and may generalise well beyond their regions of known occurrence [4]. National surveys after the September 11 attacks showed that around half of the U.S. adult population felt that they or their family were at personal risk of harm in a terrorist attack [5,6]. Perceived threat is a factor of interest to public health and emergency planners, as it plays a key role in motivating individuals to engage in appropriate protective behaviours, including terrorism-related responses [7,8]. At the same time, exaggerated personal fears can also have adverse health and socio-economic effects, including movement restrictions, avoidance of transport modes, substance abuse and community conflict [9-12].

Specific demographic groups have been found to experience particular vulnerability to terrorism-related threats. Although males and members of majority ethnic groups commonly report limited perceived risk and post-incident functional impacts (the so-called 'white male effect'), meta-analyses show that middle age, female gender and ethnic minority status are factors associated with greater impacts following both natural disasters and terrorist incidents [13]. Studies following September 11 and the London 2005 transport bombings identified similar risk groups [9,14]. In countries such as Canada and Australia, which have experienced relatively little domestic terrorism, there is evidence of similar vulnerabilities within these populations [15-17]. Australian research in 2007 identified high levels of concern and changes in living particularly among those of ethnic minority status [16]. Moreover, the specific pattern of results was suggestive of perceived secondary threats within the general community, rather than the direct risks of terrorist incidents per se.

While it is important to understand population effects after a major terrorist incident, communities with histories of lower exposure but high notional risk also warrant research attention. Commenting on Canadian research, Lee et al [2] argue that information about the way individuals perceive and respond to such threats, prior to their occurrence, can be used in the development of strategies aimed at preparing for terrorism. Such initiatives, they argue, represent a valuable shift from reactive to proactive emergency management. In this vein, Australian population data were gathered in 2007 as part of a surveillance process similar to those occurring in other countries [8]. The aim of this study is to use pooled data from surveys conducted in 2007 and 2010 to determine socio-demographic and health factors associated with higher perceived risk of terrorism and evacuation response intentions, and to examine changes over time.

## Methods

A search of existing survey instruments identified a Canadian study on terrorism perceptions and anticipated responses, which was used as a primary reference for the current survey [15]. Questions on threat likelihood, concern for self/family, and protective behaviours (i.e. willingness to comply with evacuation requests) were adapted, with permission, by the current authors. Perceived likelihood and vulnerability/concern are commonly used indicators of 'threat perception' (i.e. serious and likely threat to health) and have been shown to predict the adoption of a range of health protective behaviours [7,8]. All survey protocols and procedures were approved by the University of Western Sydney ethics committee (protocol no. H7143).

The first survey was conducted as part of the New South Wales Population Health Survey between 22 January and 31 March 2007. The second was administered as a stand-alone survey between 29 October 2009 and 20 February 2010. Both surveys were conducted at the NSW Health Survey program using a Computer Assisted Telephone Interview (CATI) system [18]. The target population for both surveys was all residents aged 16 years and over, living in NSW and stratified by geographical region. Details of the validation of the original question set and its CATI administration have been previously reported [19].

### Measurements

A five question module was developed for the 2007 survey to measure terrorism threat perception variables and willingness to evacuate [17]. These same five items were also used as outcome variables in the 2010 survey. As the field test data had indicated high assumed knowledge regarding the concept of terrorism and presumptions this typically involved bombings or shootings (i.e. 'conventional' terrorism), a specific definition of terrorism was not outlined in the survey preamble.

Threat perception and response intentions were measured on a five-point Likert scale from 1 ('not at all') to 5 ('extremely'). The response categories were dichotomised into the indicators of interest (e.g. 'high concern') to address the research questions pertaining to high risk perception and response intentions. Responses of "don't know" and refused were excluded. In relation to the four hypothetical questions (i.e. perceived likelihood of a terrorist attack in Australia; concern that self or family would be directly affected; willingness to comply with evacuation of home; willingness to comply with evacuation of workplace or public facility), the two upper responses of 'very' and 'extremely' were combined to form the indicators of interest (e.g. high concern). For the non-hypothetical, behavioural question "how much have you changed the way you live your life because of the possibility of a terrorist attack", the responses 'a little', 'moderately', 'very much' and 'extremely' were combined into the indicator of interest; 'changed living'.

The socio-demographic factors that were examined for their associations with threat perception and willingness to evacuate were: age, marital status; children under 16 years of age living in household; residential location (urban or rural, as determined by Area Health authority); being born in Australia; speaking a language other than English at home (LOTE); highest educational qualification, employment status and pre-tax household income. The threat perception and intention variables were also assessed against current physical and psychological wellbeing, specifically; self-rated health status and current psychological distress, as measured by the 10-item Kessler Psychological Distress Scale (K10). Scores on the K10 range from 10-50, with ≥ 22 being considered 'high' psychological distress [20].

### Statistical analysis

The data from both surveys were weighted to be representative of the target population and to adjust for probability of selection and differing non-response rates among males and females and different age groups, and in a manner consistent with established NSW Health population health survey program methodology [21]. The data sets have also been shown to be representative of the national population in terms of its key demographic characteristics and this comparison has been previously reported [19]. However, the potential for differences in terrorism threat perceptions to be geographically-driven means that it would not be appropriate to generalise these findings to all Australian States. Data analysis was performed using the "SVY" commands of Stata version 10.0 (Stata Corp, College Station, TX, USA), which allowed for adjustments for sampling weights. The Taylor series linearization method was used in the surveys when estimating confidence intervals around prevalence estimates. A chi-squared test was used to test the significance of associations.

The 2007 and 2010 data were combined into a single data set. A logistic regression generalised linear latent and mixed models (gllamm) analysis, [22] which adjusts for the effect of clustering (local government areas), was conducted using a stepwise backwards method in order to determine the independent variables (socio-demographic and health factors) significantly associated with the terrorism threat perception and response intent variables. Potential confounders were adjusted for as part of the analysis, with these consisting of all the independent variables previously noted. Also adjusted for as part of the evacuation analysis were the two threat perception variables (likelihood and concern). These were included as independent variables in this analysis in order to specifically explore these associations. The odds ratios with 95% confidence intervals were calculated in order to assess the adjusted risk of the independent variables, and those with p < 0.05 were retained in the final model.

## Results

The samples for the 2007 and 2010 surveys were 2,081 and 2,038 respectively, yielding a total sample of 4,119 respondents for the pooled analysis. The survey response rates were determined in accordance with NSW Health Survey methodology and calculated as total completed interviews of eligible participants, divided by the combined total of completed interviews and refusals [18]. For the 2007 survey a total of 17,439 call attempts were made, yielding 3,372 eligible participants (2,081 completions and 1,191 refusals) and a response rate of 63.6%. The 2010 survey involved 18,300 call attempts, which yielded 3548 eligible participants (2,038 completions and 1510 refusals) and a response rate of 57.4%.

Results of the 2010 survey showed that 32.5% of the population thought a terrorist attack was very or extremely likely in Australia, 38.4% were very or extremely concerned that they or their family could be directly affected by such an incident and 27.1% had made some (small to extreme) level of change to the way they lived their life in response to the perceived risk of a terrorist incident. These findings compared with prevalence rates in the 2007 survey of 30.3%, 42.5% and 26.4% respectively. High willingness to evacuate from a home or a workplace/public facility in the context of a potential terrorist threat was reported by 75.0% and 88.0% of respondents respectively in the 2010 survey and 67.4% and 85.2% in the 2007 survey.

**Figure 1:**
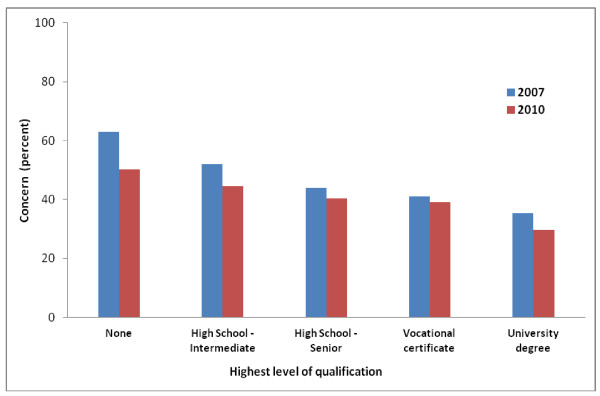
**Prevalence of high concern for self and family by educational status in 2007 and 2010**.

With regard to the threat perception (likelihood, concern) and changed living factors, concern showed the greatest change over the two surveys. When examined by educational category, Figure 1 shows that the prevalence of reported high concern was lower across all categories in 2010 compared to 2007, with greater reductions occurring for respondents with the lowest levels of formal education.

The results of the multivariate analysis for the threat perception/changed living factors are presented in Table 1. These results show that in comparison with 2007, Australians in 2010 were significantly more likely to believe that a terrorist attack would occur in Australia (AOR = 1.24, p = 0.008) but felt less concerned that they would be directly affected by such an incident (AOR = 0.65, p < 0.001).

**Table 1:** Multilevel modelling of terrorism likely, concern and changed living - unadjusted & adjusted Odds Ratios

Independent variable	Terrorist attack likely				Concern self or family directly affected				Changed way of living due to possibility of terrorist attack			
	
	Unadjusted		Adjusted		Unadjusted		Adjusted		Unadjusted		Adjusted	
	
	OR	(95%CI)	AOR	(95%CI)	OR	(95%CI)	AOR	(95%CI)	OR	(95%CI)	AOR	(95%CI)
**Year of survey**												
2007	1.00		1.00		1.00		1.00		1.00		1.00	
2010	1.20	(1.05,1.36)	1.24	(1.06,1.45)	0.69	(0.61,0.78)	0.65	(0.55,0.75)	1.09	(0.95,1.26)	1.10	(0.89,1.35)
**Gender**												
Male	1.00				1.00		1.00		1.00		1.00	
Female	1.01	(0.88,1.15)			1.75	(1.53,1.99)	1.77	(1.51,2.07)	1.28	(1.10,1.49)	1.31	(1.09,1.58)
**Location**												
Urban	1.00				1.00		1.00		1.00		1.00	
Rural	1.34	(1.16,1.55)			0.80	(0.69,0.94)	0.66	(0.56,0.77)	0.71	(0.61,0.82)	0.83	(0.69,1.00)
**High Psychological** **Distress**												
No	1.00		1.00		1.00		1.00		1.00		1.00	
Yes	1.53	(1.26,1.86)	1.46	(1.19,1.78)	1.62	(1.33,1.97)	1.63	(1.32,2.00)	1.54	(1.25,1.91)	1.70	(1.34,2.16)
**Age in categories**												
16-24	1.00		1.00		1.00		1.00		1.00			
25-34	2.21	(1.50,3.25)	2.48	(1.57,3.92)	1.33	(0.95,1.85)	1.60	(1.07,2.40)	1.13	(0.78,1.63)		
35-44	2.78	(1.93,4.00)	2.92	(1.90,4.47)	0.94	(0.69,1.29)	1.12	(0.76,1.63)	1.28	(0.91,1.80)		
45-54	3.04	(2.14,4.32)	2.89	(1.90,4.38)	1.28	(0.95,1.72)	1.33	(0.92,1.92)	1.30	(0.94,1.81)		
55-64	2.85	(2.01,4.04)	2.50	(1.65,3.79)	1.44	(1.07,1.93)	1.47	(1.02,2.12)	1.09	(0.78,1.51)		
65-74	2.63	(1.84,3.75)	2.42	(1.59,3.68)	1.56	(1.16,2.11)	1.58	(1.09,2.28)	0.74	(0.53,1.04)		
75+	2.17	(1.49,3.16)	1.61	(1.03,2.51)	1.70	(1.24,2.34)	1.81	(1.22,2.71)	0.62	(0.43,0.90)		
**Children in household**												
No	1.00				1.00				1.00			
Yes	1.25	(1.10,1.43)			0.81	(0.72,0.92)			1.17	(1.01,1.35)		
**Born in Australia**												
No	1.00				1.00				1.00			
Yes	1.32	(1.12,1.55)			0.91	(0.78,1.06)			0.65	(0.55,0.77)		
**Speak language other than English**												
No	1.00		1.00		1.00		1.00		1.00		1.00	
Yes	0.53	(0.41,0.68)	0.52	(0.39,0.70)	1.49	(1.20,1.85)	1.57	(1.21,2.04)	2.65	(2.12,3.29)	2.47	(1.88,3.25)
**Highest qualification**												
University degree	1.00		1.00		1.00		1.00		1.00			
Vocational certificate	1.58	(1.31,1.90)	1.56	(1.26,1.92)	1.63	(1.35,1.96)	1.85	(1.49,2.30)	0.90	(0.74,1.11)		
High School (Senior)	1.15	(0.93,1.43)	1.37	(1.06,1.76)	1.57	(1.27,1.94)	1.62	(1.26,2.09)	0.98	(0.78,1.24)		
High School (Inter)	1.56	(1.30,1.87)	1.74	(1.40,2.15)	2.38	(1.98,2.86)	2.52	(2.02,3.14)	1.05	(0.86,1.28)		
None	2.17	(1.71,2.75)	2.25	(1.70,2.99)	2.89	(2.28,3.66)	2.95	(2.21,3.93)	1.01	(0.77,1.31)		
**Work (paid or unpaid)**												
No	1.00				1.00				1.00			
Yes	0.95	(0.84,1.09)			0.71	(0.63,0.81)			1.14	(0.99,1.32)		
**Household income**												
< $20 k	1.00				1.00				1.00			
$20-40 k	1.08	(0.88,1.32)			0.84	(0.68,1.02)			1.07	(0.85,1.35)		
$40-60 k	0.97	(0.78,1.22)			0.70	(0.56,0.87)			1.11	(0.87,1.43)		
$60-80 k	0.84	(0.65,1.07)			0.71	(0.55,0.90)			1.01	(0.76,1.33)		
> $80 k	0.74	(0.60,0.90)			0.53	(0.44,0.65)			1.13	(0.91,1.14)		
**Marital status**												
Married	1.00				1.00				1.00			
Widowed	0.87	(0.70,1.06)			1.55	(1.28,1.89)			0.78	(0.62,0.99)		
Separated/divorced	1.24	(1.03,1.49)			0.95	(0.79,1.15)			0.93	(0.75,1.15)		
Never married	0.69	(0.58,0.82)			0.81	(0.69,0.97)			0.92	(0.76,1.11)		
**Good self-rated health**												
Yes	1.00				1.00				1.00		1.00	
No	1.29	(1.08,1.53)			1.24	(1.05,1.48)			0.94	(0.77,1.14)	0.78	(0.63,0.98)

Note: 95% confidence intervals (CI) that include 1.00 indicate a non significant result Psychological distress was measured using the K10. Values range from 10-50, with ≥ 22 considered 'high' psychological distress.

**Figure 2:**
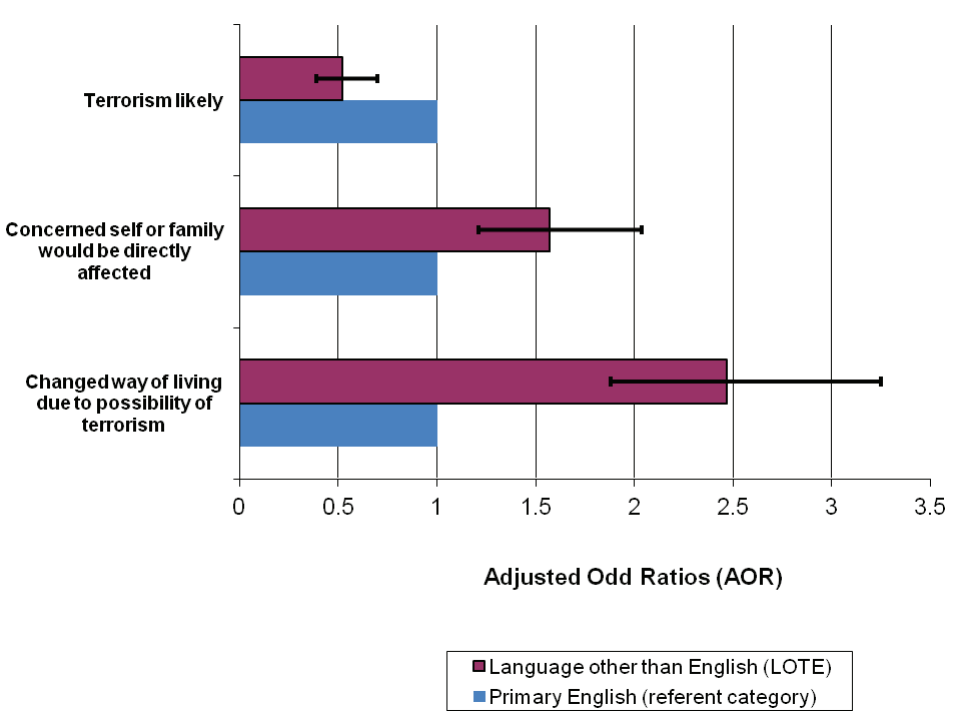
**Adjusted Odd Ratios of terrorism likely, concern and changed living by primary language group**.

In relation to the demographic variables, Australians with no formal educational qualifications were significantly more likely (AOR = 2.25, p < 0.001) to report that they perceived a terrorist attack as being very/extremely likely to occur, compared to those with university level qualifications; as were middle aged respondents (45-54 years) compared to young people (16-24 years) (AOR = 2.89, p < 0.001). Respondents who spoke a language other than English at home (LOTE) were significantly less likely to report a terrorist attack as being very/extremely likely to occur (AOR = 0.52, p < 0.001). The Adjusted Odd Ratios for LOTE respondents regarding threat perception and changed living are presented in Figure 2.

People living in urban areas were significantly more likely to be very/extremely concerned that they or their family would be directly affected in the event of a terrorist attack (AOR = 0.66, p < 0.001) as were: females (AOR = 1.77, p < 0.001); those with no formal educational qualifications, compared to those with university qualifications (AOR = 2.95, p < 0.001) and LOTE respondents (AOR = 1.57, p < 0.001). Australians who spoke a language other than English at home were significantly more likely to report having made changes in the way they lived due to the possibility of terrorism (AOR = 2.47, p < 0.001). Other positive predictors of this indicator were female gender (AOR = 1.31, p = 0.004) and urban residency (AOR = 0.83, p = 0.054). Respondents with poor self-rated health were significantly less likely to have changed their way of living due to concerns about terrorism (AOR = 0.78, p = 0.033).

With regards to the physical and psychological health status variables, positive findings within the multivariate analysis were observed only in relation to the latter item. High psychological distress was associated with high perceived terrorism likelihood (AOR = 1.46, p < 0.001), high concern that self or family would be directly affected by an attack (AOR = 1.63, p < 0.001) and higher likelihood of having made some changes in living due to this threat (AOR = 1.70, p < 0.001).

The results of the protective behaviours analysis are presented in Table 2 and show that, compared with 2007, Australians in 2010 were significantly more likely (AOR = 1.54, p < 0.001) to report being very/extremely willing to evacuate their home during a terrorism related emergency. This was also the case for the combined home/work place or public facility indicator (AOR = 1.53, p < 0.001).

**Table 2:** Multilevel modelling of willingness to evacuate home and office/public facility - unadjusted & adjusted Odd Ratios

Independent variable	Willing to evacuate home				Willing to evacuate office/public facility				Willing to evacuate home and office/public facility			
	
	Unadjusted		Adjusted		Unadjusted		Adjusted		Unadjusted		Adjusted	
						
	OR	(95%CI)	AOR	(95%CI)	OR	(95%CI)	AOR	(95%CI)	OR	(95%CI)	AOR	(95%CI)
**Year of survey**												
2007	1.00		1.00		1.00		1.00		1.00		1.00	
2010	1.41	(1.23,1.61)	1.54	(1.33,1.78)	1.31	(1.10,1.57)	1.12	(0.85,1.49)	1.41	(1.23,1.61)	1.53	(1.33,1.76)
**Gender**												
Male	1.00		1.00		1.00		1.00		1.00		1.00	
Female	1.45	(1.27,1.67)	1.40	(1.21,1.62)	1.61	(1.34,1.92)	1.61	(1.30,2.00)	1.41	(1.23,1.61)	1.36	(1.17,1.57)
**Location**												
Urban	1.00				1.00				1.00			
Rural	0.90	(0.78,1.04)			0.90	(0.74,1.09)			0.90	(0.79,1.04)		
**Age in categories**												
16-24	1.00		1.00		1.00		1.00		1.00		1.00	
25-34	1.20	(0.84,1.72)	1.02	(0.70,1.48)	1.35	(0.85,2.14)	1.19	(0.66,2.13)	1.20	(0.85,1.70)	1.03	(0.72,1.47)
35-44	1.43	(1.02,2.00)	1.20	(0.85,1.71)	1.70	(1.09,2.63)	1.27	(0.73,2.23)	1.44	(1.04,1.99)	1.20	(0.85,1.68)
45-54	1.36	(0.99,1.87)	1.14	(0.82,1.58)	1.78	(1.17,2.71)	1.27	(0.75,2.16)	1.44	(1.06,1.96)	1.21	(0.88,1.67)
55-64	1.07	(0.79,1.47)	0.94	(0.68,1.30)	1.55	(1.03,2.33)	1.26	(0.75,2.11)	1.14	(0.84,1.54)	0.99	(0.72,1.36)
65-74	0.67	(0.49,0.91)	0.65	(0.46,0.90)	0.80	(0.54,1.18)	0.82	(0.49,1.36)	0.67	(0.50,0.91)	0.64	(0.47,0.89)
75+	0.42	(0.30,0.58)	0.39	(0.27,0.55)	0.48	(0.32,0.71)	0.46	(0.27,0.77)	0.41	(0.30,0.56)	0.39	(0.27,0.55)
**Children in household**												
No	1.00				1.00		1.00		1.00			
Yes	1.45	(1.27,1.66)			1.57	(1.31,1.88)	1.35	(1.01,1.79)	1.46	(1.28,1.67)		
**Born in Australia**												
No	1.00				1.00				1.00			
Yes	1.02	(0.87,1.21)			1.30	(1.06,1.61)			1.07	(0.91,1.25)		
**Speak language other** **than English**												
No	1.00				1.00		1.00		1.00			
Yes	1.12	(0.88,1.43)			0.62	(0.47,0.82)	0.52	(0.36,0.73)	1.02	(0.81,1.29)		
**Highest qualification**												
University degree	1.00				1.00		1.00		1.00			
Vocational certificate	0.84	(0.69,1.03)			0.90	(0.68,1.19)	1.26	(0.95,1.68)	0.85	(0.70,1.04)		
High School (Senior)	0.68	(0.54,0.84)			0.57	(0.43,0.77)	1.53	(0.09,2.16)	0.69	(0.56,0.86)		
High School (Inter)	0.74	(0.61,0.90)			0.63	(0.48,0.81)	2.35	(1.52,3.62)	0.72	(0.48,0.87)		
None	0.65	(0.51,0.83)			0.49	(0.36,0.68)	2.45	(1.73,3.49)	0.61	90.48,0.78)		
**Work (paid or unpaid)**												
No	1.00		1.00		1.00				1.00		1.00	
Yes	1.78	(1.56,2.04)	1.28	(1.10,1.56)	2.00	(1.67,2.40)			1.80	(1.58,2.06)	1.31	(1.11,1.55)
**Household income**												
< $20 k	1.00				1.00				1.00			
$20-40 k	1.43	(1.16,1.77)			1.32	(1.01,1.72)			1.37	(1.11,1.68)		
$40-60 k	1.56	(1.23,1.98)			1.63	(1.20,2.22)			1.53	(1.21,1.92)		
$60-80 k	1.62	(1.25,2.10)			2.78	(1.87,4.15)			1.76	(1.37,2.28)		
> $80 k	2.21	(1.79,2.73)			2.93	(2.17,3.95)			2.24	(1.83,2.75)		
**Marital status**												
Married	1.00				1.00				1.00			
Widowed	0.56	(0.45,0.88)			0.62	(0.48,0.80)			0.55	(0.45,0.67)		
Separated/divorced	0.98	(0.80,1.20)			1.05	(0.79,1.38)			0.97	(0.80,1.19)		
Never married	0.97	(0.81,1.16)			0.80	(0.64,1.01)			0.96	(0.80,1.14)		
**Good self-rated health**												
Yes	1.00				1.00				1.00			
No	0.81	(0.68,0.97)			0.80	(0.64,1.01)			0.84	(0.70,1.00)		
**High Psychological Distress**												
No	1.00				1.00				1.00			
Yes	1.13	(0.91,1.41)			0.83	(0.63,1.09)			1.06	(0.86,1.31)		
**Terrorism likely**												
No	1.00		1.00		1.00		1.00		1.00		1.00	
Yes	1.49	(1.29,1.72)	1.29	(1.10,1.50)	2.16	(1.75,2.67)	1.94	(1.50,2.50)	1.56	(1.35,1.80)	1.36	(1.17,1.59)
**Concerned self/family** **directly affected**												
No	1.00		1.00		1.00		1.00		1.00		1.00	
Yes	1.71	(1.48,1.98)	1.79	(1.53,2.10)	1.70	(1.40,2.06)	1.85	(1.45,2.36)	1.69	(1.47,1.95)	1.75	(1.50,2.04)

Note: 95% confidence intervals (CI) that include 1.00 indicate a non significant result Psychological distress was measured using the K10. Values range from 10-50, with ≥ 22 considered 'high' psychological distress.

Those without formal educational qualifications were significantly more likely to report high willingness to evacuate public facilities (AOR = 2.45, p < 0.001). Females reported higher willingness with both single indicators and the combined indicator (AOR = 1.36, p < 0.001). Being over 75 years of age was associated with significantly lower willingness on all indicators (combined indicator AOR = 0.39, p < 0.001), while those aged 65-74 years reported lower willingness regarding home evacuation and the combined indicator (AOR = 0.64, p = 0.008). LOTE respondents reported significantly lower willingness to evacuate public facilities (AOR = 0.52, p < 0.001).

The two threat perception variables (likelihood and concern respectively) were associated with evacuation intent in all categories, these being: home evacuation (AOR = 1.29, p = 0.002) (AOR = 1.79, p < 0.001), offices or public facilities (AOR = 1.94, p < 0.001) (AOR = 1.85, p < 0.001) and the combined indicator (AOR = 1.36, p < 0.001) (AOR = 1.75, p < 0.001). Overall, concern for self and family was more strongly associated with evacuation intent than was perceived likelihood of terrorism.

## Discussion

The current results indicate that between 2007 and 2010 there has been a small but significant increase in the perceived likelihood that a terrorist incident will occur in Australia. At around one third of the population perceiving high likelihood of an attack, these findings are consistent with results from the 2007 Australian Survey of Social Attitudes (34.8%) but lower than rates observed in the Australian Wellbeing Survey [23,24]. The latter survey series showed perceived likelihood peaked and then declined after both the 2002 and 2005 Bali bombings, probably the two most significant terrorist incidents to have directly affected Australians. The latter reduction occurred at a faster rate, leading the authors to conclude that the initial incident exposure had supported community adaptation to the subsequent event. The specific pattern of the current findings; reduction in personal concern/vulnerability against a small rise in likelihood, also suggests a general habituation to this threat has occurred over the 2007-2010 period.

As the 2010 survey was conducted over the more extended period of four months, it was necessary to test for potential time effects that may have affected those findings. While no attacks directly affecting Australians occurred during either survey, the widely reported 'underwear bomber' incident occurred on a Chicago-bound plane on December 25, 2009. This was the midpoint of the second survey and a high period of travel during the Australian summer holiday season. There was sufficient statistical power to examine survey response by month and this analysis confirmed that there were no significant differences, by month, in relation to perceived likelihood (p = 0.263), concern (p = 0.625) or living changes (p = 0.5).

While habituation to terrorism threat has been reported in relation to ongoing political violence in Israel [25,26] and after large-scale single incidents [14], the current findings indicate that similar processes may occur within populations which have had limited direct exposure. This tendency for individuals to adapt to stressors over time and return to 'baseline' arousal is an increasingly recognised element of community recovery and resilience processes. Moreover, these functional 'trajectories' may be optimised (e.g. pre-emptive community education/resilience programs) or potentially undermined [27]. For example, Tucker suggests that post 9/11 media and government messaging about threats may have impeded habituation by providing constant fear-based reminders [28]. Conversely, effective terrorism risk communication may help the public to regain its 'basal security' by clearly explaining the nature of the threat, its likelihood and current management, and addressing its unknown/dreaded aspects [29]. Achieving this lower state of arousal means that subsequent threat information is likely to be assessed in qualitatively different ways, which are more likely to promote adaptive outcomes (e.g. reduced avoidance behaviours) [4].

Contextual factors may also be important mediators of habituation. This may be seen in the current finding that urban residents were significantly less likely than rural residents to report high perceived incident likelihood, despite a dominant worldwide trend towards urban forms of terrorism. Goodwin et al [30] found that those living in urban London reported lower perceived likelihood of attack than those in suburban and rural areas, a process they suggest is related to cognitive dissonance (i.e. minimising the clash arising from the desire for safety and the simultaneous choice of a 'high risk' habitat). While such factors may promote habituation, it is also important to consider whether the currently observed reductions in concern represent adaptive or more maladaptive forms of habituation (e.g. perceived invulnerability in the absence of attacks). Importantly, co-occurring increases in terrorism likelihood and evacuation willingness suggest that lower reported concern, in this context, does not equate with complacency and that the public could be readily mobilised were alert levels to increase.

The period between the 2007 and 2010 surveys was marked by several terrorism-related incidents that received wide media coverage in Australia. These included the Mumbai attacks and the 2008 trial of twelve 'home-grown' terrorists who had planned the bombing of major sporting complexes in Melbourne. In July and August 2009, the period prior to the second survey, there was also reporting of the Jakarta hotel bombing and the uncovered plot to attack the Holsworthy Army Barracks in Sydney. It is likely that coverage of these incidents has maintained terrorism as a 'front of mind' threat within the Australian population, possibly keeping the perceived likelihood of an incident relatively steady during this period. Despite such awareness, only about one quarter of respondents reported any discernable change in the way they live due to this threat; a rate similar to that observed in U.S. population surveys [31].

The observed reduction in level of concern for self or family may be due to a number of factors. One possibility is that through an increased awareness of 'real' terrorist plots within Australia, respondents may have realised that they or their family would not have been directly impacted, thus altering their appraisal of the personal risks of such events. The specific nature of recent threats may have also been a factor, that is, gun-related sieges typified by the 2008 Mumbai attacks and the planned Holsworthy assault. Incidents of this kind may actually be more culturally 'familiar' than other scenarios (e.g. co-ordinated suicide bombings, chemical attacks) and provide clearer reference points against which people can assess their own vulnerability [4]. This may have been a contributing factor in the large reported increases in evacuation willingness between the 2007 and 2010 surveys. Paradoxically, such threats may also lack the dread and high-novelty elements known to inflate personal risk estimates, [3] thereby contributing to reduced levels of concern.

The pooled analysis highlights that middle age, female gender and lower education; demographic factors identified with higher perceived terrorism risk in post incident settings, [5,8,9] are also common to western settings outside that event context. This information can assist public health and emergency planners in these latter settings as these potential 'lead' groups may engage more readily with risk communication initiatives, including appropriate vigilance raising and alerts. Female respondents may be an important case in point. While gender is a commonly identified 'risk factor' in the threat perception literature, the current results show that there is not an undifferentiated bias in females' reports i.e. they do not perceive terrorism as more likely but do report greater concern. As such, women may see the 'reality' of this risk in much the same way as men but also experience greater concern for people in general, irrespective of the threat source. Counter to interpretations which tend to invoke traditional gender models, [32] these findings highlight that women could be easier to engage and may be more effective allies in terrorism risk mitigation efforts.

Younger people (16-24 years) routinely show lower levels of threat perception (i.e. incident likelihood and concern). While a potential positive in itself, preparedness for any heightened threat may warrant dedicated risk messaging for this group, including the possible use of social media platforms [33]. An inverse correlation was also observed between reported concern and level of education (Figure 1). Similar previous findings have been interpreted as reflecting education-related appraisal capacities or, alternatively, the associated availability of financial or other resources that may 'buffer' against potential threat [16]. While education is a factor of known importance in the risk communication field generally [4] it has received little detailed analysis as an element of terrorism preparedness. In this vein, it is notable that those with the greatest reduction in concern between 2007 and 2010 were those with the lowest levels of formal education.

A notable finding was that LOTE respondents reported significantly lower levels of terrorism likelihood, but significantly higher levels of concern and perceived changes in the way they lived due to terrorism threat. While this pattern in the pooled results is broadly consistent with the 2007 findings [16], the pooled analysis highlights a greater dissonance in relation to these variables (Figure 2). As noted with female respondents, this may similarly reflect a higher concern for family and others in general, possibly associated with cultural and out-group bonding processes [16]. A further interpretation may be that heightened community anxiety about terrorism could be associated with increased marginalisation of visible minority groups. For such groups, it is possible that wider societal reactions represent a more pervasive threat to their wellbeing than potential terrorist acts. In terrorism affected countries, culture, appearance and religion have been found to be strong predictors of terrorism-related distress and appear to reflect increased stigmatisation of these groups [9].

While it must be seen that LOTE represents a broad indicator within the current study, this pattern of results could similarly reflect social 'splintering' around this issue within Australia [34]. This is consistent with qualitative research with minority groups highlighting perceptions that terrorism issues are frequently used as a rationale for cultural/religious and race-related harassment by members of the wider Australian community. This is particularly the case after specific incidents have occurred [12]. While those of Arab and Muslim background have been disproportionately affected, vilification of other ethnic and religious groups has also been documented [12]. Such findings present significant social policy challenges regarding the effective promotion of cultural tolerance and social inclusion; including the nature and timing of specific initiatives [35]. While these are important outcomes in their own right, there is growing awareness that actively increasing social engagement may also be a key counter-terrorism strategy; since such environments may be less conducive to the radicalisation of vulnerable individuals [35,36].

The consistent association between psychological distress and terrorism likelihood/concern was not observed in the 2007 study and appears to indicate a broad increase in perceived terrorism threat within this sub-group. This may be due to the frequency or particular nature of more recent incidents coupled with a greater sensitivity to them. Australian research has shown that strong belief in the likelihood of an attack is associated with low personal wellbeing and suggests that, for such groups, the practice of issuing 'blanket alerts' may be counter-productive [24]. Conversely, there is evidence that experimentally induced mood (i.e. fear) increases terrorism risk estimates and motivation towards protective behaviours, [37] albeit this may represent a qualitatively different mood state and context. In the current analysis for example, higher distress was not associated with higher evacuation intent and may indicate a more complex interaction between negative affect and safety appraisals in these scenarios.

The willingness of respondents to evacuate from their homes during a potential terrorist incident was significantly higher in 2010 compared to 2007. This was not the case for evacuation relating to offices/public facilities, although this may be due to a ceiling effect in the earlier study (i.e. 85% reported high compliance). Consistent with health protection motivation theories, higher perceived incident likelihood and concern were associated with higher evacuation intent in all categories [7]. This supports the limited available evidence for this relationship in post incident settings [8], while also showing that it is not limited to these contexts. The finding that concern for others was a stronger predictor of intent than was likelihood also highlights the potential value of a new approach to risk communication in this area; one that engages people around the protection of loved ones and others, rather than fear of terrorism occurrence per se [15].

The increased willingness regarding home evacuation is somewhat unusual in that people typically show greater reluctance to leave their homes during threatened emergencies [38]. This result may be due to a stronger perception during the second survey period that scenarios warranting such evacuations could realistically occur. As noted, there has been wide-spread coverage of terrorism-related activity during this period, including the deaths of Australians during the Jakarta and Mumbai incidents. The latter was a protracted urban assault that received intense coverage and was notable for its 'low tech' but highly disruptive method. It has been argued that qualitatively different incidents like this may have 'signal potential' in that they can promote widespread changes in perceptions and specific protective behaviours [39].

### Limitations

The current study has several limitations. While the 2007 and 2010 survey response rates of 63.6% and 57.4% respectively compare favourably with similar population surveys, [9] they had the potential to introduce a response bias in relation to the current results. As noted, this was addressed by introducing weightings to adjust for probability of selection and for differing non-response rates among males and females and different age groups. While NSW residents make up around one third of the Australian population and the weighted NSW sample is consistent with national population demographics, [19] potential regional variations in terrorism threat perception mean that the current findings cannot be generalised to all Australian States.

The aim of this study is to determine socio-demographic and health factors associated with terrorism threat perception and response intentions using a large, pooled data set. While the sample size is a strength of this study, its repeated cross-sectional design captures only a snapshot view of these frequencies at two different time points and no firm conclusions can be made regarding causes. The current findings raise important questions regarding demographic groups with potentially greater vulnerability, particularly during periods of heightened perceived threat and in the aftermath of possible future incidents. Qualitative research being conducted by the current authors will provide further insight into the underlying issues affecting these outcomes, and whether these have implications for social policy and incident response planning.

## Conclusion

The current findings appear to show a pattern of population-level habituation to the threat of terrorism in Australia during the study period 2007-2010. Rather than simply reflecting reduced preoccupation over time, the pattern of findings suggest an increased appreciation of specific risk scenarios, including the relative risks that individuals may face. Key demographic groups remain disproportionately concerned, notably migrant groups and those with lower education. The dissonance observed in pooled findings regarding Australians of migrant background was more pronounced than in our earlier analysis. It is also consistent with qualitative data showing wider socio-cultural concerns associated with this issue. Amid recent challenges in Australia to the ethos of multiculturalism, public messaging that supports cultural diversity may also play an important role in reducing specific community discord that has centred on terrorism fears.

## Competing interests

The authors declare that they have no competing interests.

## Authors' contributions

GS and BR conceived the idea and designed the study. GS carried out the statistical analysis and wrote the manuscript. All authors made contributions to the interpretation of results and revised the manuscript for important intellectual content. All authors read and approved the final version of the manuscript.

## Pre-publication history

The pre-publication history for this paper can be accessed here:

http://www.biomedcentral.com/1471-2458/11/797/prepub
